# A Proteomic View at T Cell Costimulation

**DOI:** 10.1371/journal.pone.0032994

**Published:** 2012-04-23

**Authors:** Rudolf Lichtenfels, Gunter Rappl, Andreas A. Hombach, Christian V. Recktenwald, Sven P. Dressler, Hinrich Abken, Barbara Seliger

**Affiliations:** 1 Institute of Medical Immunology, Martin-Luther-University Halle-Wittenberg, Halle, Germany; 2 Center for Molecular Medicine Cologne (CMMC) and Tumor Genetics Section, Clinic I Internal Medicine, University Cologne, Cologne, Germany; Institute of Pathology, Germany

## Abstract

The “two-signal paradigm” in T cell activation predicts that the cooperation of “signal 1,” provided by the T cell receptor (TCR) through engagement of major histocompatility complex (MHC)-presented peptide, with “signal 2″ provided by costimulatory molecules, the prototype of which is CD28, is required to induce T cell effector functions. While the individual signalling pathways are well understood, little is known about global changes in the proteome pattern during TCR/CD28-mediated activation. Therefore, comparative 2-DE-based proteome analyses of CD3^+^ CD69^-^ resting T cells versus cells incubated with (i) the agonistic anti-CD3 antibody OKT3 mimicking signal 1 in absence or presence of IL-2 and/or with (ii) the agonistic antibody 15E8 triggering CD28-mediated signaling were performed. Differentially regulated spots were defined leading to the identification of proteins involved in the regulation of the metabolism, shaping and maintenance of the cytoskeleton and signal transduction. Representative members of the differentially expressed protein families, such as calmodulin (CALM), glyceraldehyde-3-phosphate dehydrogenase (GAPDH), L-lactate dehydrogenase (LDH), Rho GDP-dissociation inhibitor 2 (GDIR2), and platelet basic protein (CXCL7), were independently verified by flow cytometry. Data provide a detailed map of individual protein alterations at the global proteome level in response to TCR/CD28-mediated T cell activation.

## Introduction

Activation and expansion of antigen-specific T cells are essential prerequisites for the successful mounting of specific immune responses, both at the cellular as well as the humoral level. The discovery of costimulatory molecules which act in concert with the TCR-mediated signals led to the “two signal paradigm” that naïve T cells require at least two activation signals, one by the TCR and the other by costimulatory molecules in order to initiate full T cell activation [1], [2], [3], [4]. Intensive research performed in this field over the last two decades has provided deep insights into the requirements and underlying mechanisms leading to the activation or inactivation of effector, memory and regulatory T cell subsets [1]. Engagement of the TCR with the peptide loaded MHC on the cell surface of the antigen presenting cell (APC) provides signal 1. The second signal is mediated by activating members of the costimulatory CD28 family [3], [4]. The CD28 ligand family of B7 molecules like B7-1 (CD80) and B7-2 (CD86), also expressed on activated APCs, promote proliferation, survival and differentiation of T cells into distinct T cell subsets [5], [6]. Lack of costimulatory signals causes T cell anergy [7], [8] while engagement of B7-1 or PD-L1 (B7-H1, CD274) and PD-L2 (B7-DC, CD273) with the inhibitory receptors CTLA-4 (CD152) or PD1 (CD279), respectively, induce T cell unresponsiveness and tolerance [5].

With respect to the profound impact on T cell function the downstream signaling cascades of the B7/CD28 pathway were further investigated in detail. While for the priming of CD4^+^ T cells the B7-1/B7-2-CD28 interaction is not always required [9], it is essential for primary and memory CD8^+^ T cell responses [4], [10], [11], [12]. CD28 not only provides T cell costimulation but also enhances the phosphoinositide 3-kinase (PI3K)/serine/threonine protein kinase (AKT) activity, which is dependent on the p110 delta isoform of PI3K at the immunological synapse [13]. The enhanced T cell APC interaction is supported by B7-1 dimers which is associated with the sustained accumulation of signaling molecules within TCR-CD28 microclusters [14], [15], [16]. In adition, a strong CD28-mediated co-stimulatory signal is necessary to induce proliferation of CD4^+^CD25^+^ regulatory T cells, which can not be substituted by IL-2 [17]. Moreover, microRNAs are involved in the control of T cell activation due to their costimulation dependent expression down-regulating the negative regulator phosphatase and tensin homolog (PTEN) [18]. CD28 costimulation has also a strong impact on T cell survival as demonstrated by an enhanced expression of the anti-apoptotic molecule B cell lymphoma –extra large (Bcl-xL) [19]. In addition Ca^2+^signaling-mediated activation of intracellular pathways are involved in costimulation via CD86 and CD28 interaction [20]. The clinical significance of these pathways became obvious in the response to superagonistic CD28-antibodies triggering the induction of harmful pro-inflammatory cytokine storms [21]. In contrast, the modulation of the CD28-mediated costimulation cascade or the inhibition of its inhibory receptor cytotoxic T-lymphocyte antigen 4 (CTLA4) exerted benefits in the treatment of auto-immunity, transplant rejections and tumors [6], [22], [23], [24], [25], [26]. In addition, CD28 costimulation can be used to amplify tumor-specific T cells [27] or to drive expansion of chimeric antigen receptor (CAR)-engineered T cells with redirected specificity [28]. Both strategies provide attractive options in the adoptive immunotherapy of cancer.

Despite increasing knowledge of the costimulatory pathways at the molecular level little is known about the impact of costimulation on the global protein expression pattern of T cells. Previous proteome studies [29], [30], [31], [32], [33] predominantely focused on quantitave changes of the phospho-proteome by applying metabolic labelings via stable isotope labeling of amino acids in cell culture (SILAC) or by stable-isotope iTRAQ labelings. However, these studies mainly used “bottom up”-proteomics approaches that are not able to distinguish between different protein isoforms. For instance the LC-MS approach for the analysis of components of the TCR/MHC peptide complex in murine T cells [34] or a phosphotyrosine proteomic profiling resulting in the identification of a new interaction partner of the TCR signalosome [29]. In addition peptide pull-down assays of phosphorylated adapter proteins and network analysis showed phosphorylation-dependent changes of protein-protein interactions in T lymphocytes [31], [32]. In contrast, less is known about the effects in T cells resulting upon the concerted TCR/CD28-mediated activation process [35], [36]. The impact of phytohaemagglutinin (PHA)-mediated activation on the global proteome [37] does not reflect the effects of TCR or costimulation-mediated T cell activation.

In this study a series of two-dimensional gel electrophoresis (2-DE)-based proteomic expression profilings were derived from highly purified human CD3^+^ T cells of healthy donors upon TCR, CD28 and combined TCR/CD28 stimulation. To thoroughly control T cell activation T cells were stimulated with agonistic antibodies mimicking TCR activation (OKT3) or CD28 triggering (15E8). Data were compared to TCR stimualion in presence of IL-2 that like CD28 provides anti-apoptotic, proliferative signals to pre-activated T cells. Extensive profiling led to the identification of a set of differentially expressed proteins that could be classified according to their function and cellular localization. Using this strategy global alterations in the T cell proteome were defined that can be discriminated with respect to TCR stimulation or CD28 costimulation only or to combined TCR/CD28 stimulation as well as IL-2 supplementation. Identified differentially expressed proteins are potential markers to assess the activation status of human T cells.

## Results

### The Mode of T Cell Activation Induces Characteristic Alterations in the Protein Expression Pattern

**Figure 1:**
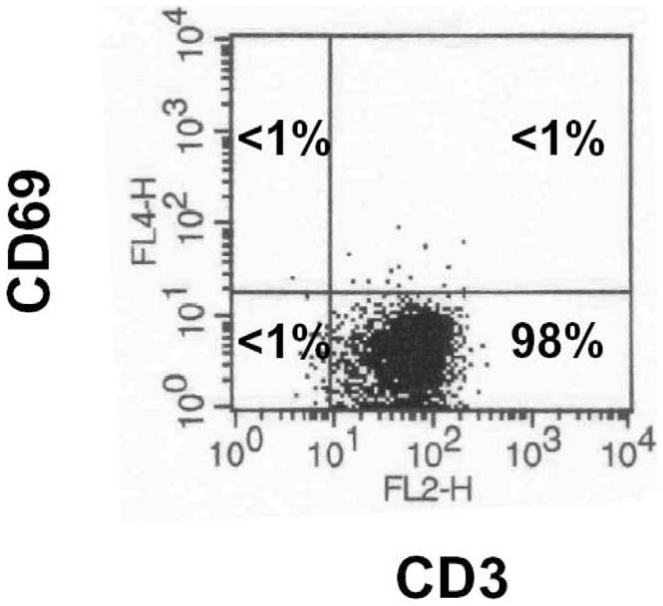
Purity of resting CD3^+^ T cell population. CD3^+^ T cells were isolated from peripheral blood by negative selection procedures using magnetic cell separation techniques. Cells were analyzed by flow cytometry using an anti-CD3 and anti-CD69 antibody. A dot plot from one representative separation is shown.

For proteomic profiling resting CD3^+^ CD69^−^ T cells were isolated to a purity of >98% by negative selection to avoid pre-stimulation (Figure 1).

**Figure 2:**
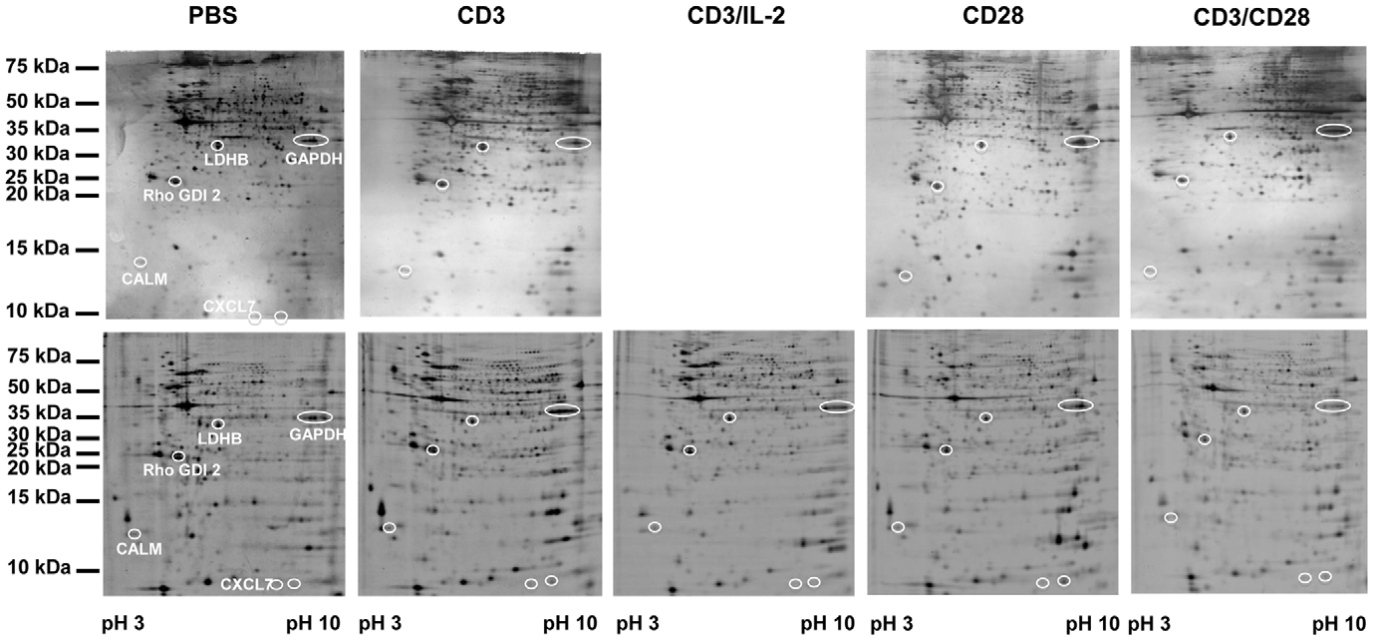
Representative protein expression profiles of T cells in response to distinct stimuli. Unstimulated (PBS) or stimulated T cells were, extracted and subsequently separated by two-dimensional gel electrophoresis in 16% T, 2.5% C polyacrylamide gels as outlined in Materials and Methods. The upper panel shows protein expression profiles obtained by separation of 150 µg total protein and visualized by silver staining, the lower panel the corresponding DIGE labelings established by separating 15 µg minimal labeled total lysate per individual sample. The stimulation procedures, the pH-gradient used in the first dimension as well as the range of the size fractionation are indicated along the axis.

Such purified T cells were either stimulated via the CD3, CD3 plus IL-2, CD28 or CD3 plus CD28 complex/receptors, respectively or by incubation with the respective agonistic antibodies, while isotype-matched antibodies served as controls. Cells were subjected to 2-DE-based proteome analyses using silver staining and/or the difference gel electrophoresis (DIGE) approach. For the latter applying a minimal labelling strategy. The 2-DE patterns found by the classical 2-DE and the DIGE technologies were similar indicating the validity of both procedures in proteome analysis. Different ways of T cell stimulation induce different patterns in the protein expression profile as representatively shown in Figure 2. Using silver stained gels 783 to 1063 spots per gel were recorded whereas minimal DIGE labellings resulted in the detection of 976 spots as summarized in Table 1.

**Table 1:** Summary of the protein expression profiling results using different staining strategies.

silver staining								
sample	CD3 vs. PBS				CD28 vs. PBS		CD3/CD28 vs. PBS	
total no. of spots	783	954			797	954	1063	954
no. of matched spots	584				581		649	
no. of upreg. spots	68				67		89	
no. of downreg. spots	72				74		93	
no. of not reg. spots	444				440		467	

DIGE					
Sample	CD3 vs. PBS	CD3/IL2 vs. PBS	CD28 vs. PBS	CD3/CD28 vs. PBS	
total no. of spots	976	976	976	976	
no. of matched spots	976	976	976	976	
no. of upreg. Spots	31	76	98	77	
no. of downreg.spots	149	206	148	96	
no. of not reg. spots	796	694	730	801	

Upper panel : analysed with Proteomweaver 4.O (Biorad, München, Germany).

Lower panel: analysed with Delta 2D (Decodon, Greifswald, Germany).

Based on the 2-DE gel analyses 97 differentially expressed spots were identified, matched to the profile of preparative gels and analysed by mass spectrometric analysis. Fiftytwo protein identities were identified in these spots. As summarized in Table S1, the profiling based on the silver stainings led to the identification of 47 differentially expressed spots. Nineteen thereof were up- and 28 spots were downregulated under different stimuation conditions. However, 8 spots contained more than one protein restricting the subset of differentially expressed proteins to 39 single target spots, representing a total number of 33 distinct protein identities (Table S2). Some proteins including glyceraldehyde 3-phosphate dehydrogenase (GAPDH), enolase alpha (ENOA) or vinculin (VINC) as well as beta-/gamma-actin (ACTB/ACTG) were detected in more than one spot suggesting that these proteins have undergone post-translational modifications.

DIGE-based profiling revealed 56 spots with increased and 75 spots with decreased expression levels in response to different stimulation conditions (Table S3). Thirty-four of the 56 up-regulated spots represent single target spots whereas the remaining 22 spots contained more than 1 protein species. Some of the 34 single target spots representing a total number of 20 protein identities were co-regulated under the individual stimulatory signals. Of the 75 spots defined as down-regulated in response to the various stimulation conditions, 55 were single target spots representing 24 different proteins (Table S3).

Again some of the differentially expressed proteins defined by this profiling strategy were represented by more than one spot identity indicating again the presence of post-translational modifications. These proteins included the protein disulfide-isomerase A3 (PDIA3), fructose-bisphosphate aldolase A (ALDOA) and VINC. Thus, the DIGE-based profiling pattern is in accordance to the pattern observed in silver stained gels.

Taking into account that some of the single target spots were partially counter-regulated under the various stimulation conditions (Table S2), a total of 37 differentially expressed proteins were identified by DIGE.

**Figure 3:**
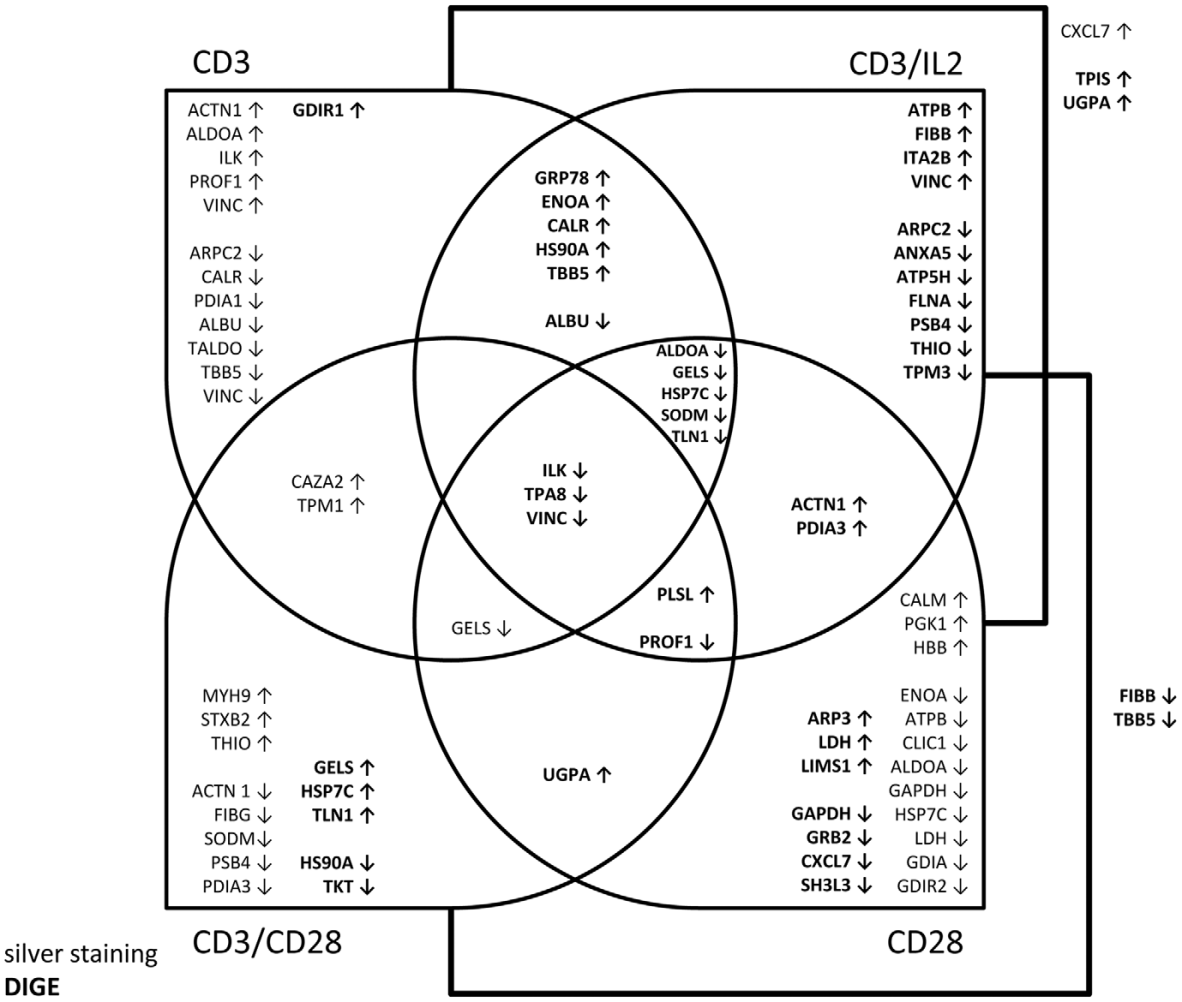
Combined profiling data for the 52 differentially expressed proteins. The venn diagramm shows the distribution of individually and shared proteins as defined by 2-DE-based proteomic profilings in response to the indicated stimulation conditions. Proteins that are either shared in response to CD3 and CD28 or CD3/IL2 and CD3/CD28 are listed next to the connecting lines. Proteins defined via silver staining are listed in plain, proteins defined by DIGE-analysis in bold. The relative regulation mode for the given protein if compared to its expression level in unstimulated T cells is indicated by arrows. Arrows pointing up indicate increased expression levels in response to stimulation, whereas downregulated proteins after flanked by arrows pointing down.

Briefly summarized, 18 of the differentially expressed proteins were defined by both profiling strategies. 15 exclusively defined by silver staining and 19 exclusively by DIGE (Table S1, Table S2 and Table S3). Proteins defined by both techniques include actin-related protein 2/3 complex subunit 2 (ARPC2), alpha-actinin-1 (ACTN1), ENOA, ATP synthase subunit beta, mitochondrial (ATPB), calreticulin (CALR), ALDOA, gelsolin (GELS), heat shock cognate 71 kDa protein (HSP7C), integrin-linked protein kinase (ILK), lactate dehydrogenase beta chain (LDHB), manganese superoxide dismutase (SODM), CXCL7, profilin-1 (PROF1), PDIA3, proteasome subunit beta type 4 (PSB4), serum albumin (ALBU), thioredoxin 1 (THIO), tubulin beta-2 chain (TBB5) and VINC. The combined profiling data for the total number of 52 differentially expressed protein identities are summarized in Figure 3 (Table S4).

### Classification of Differentially Expressed Proteins

**Figure 4:**
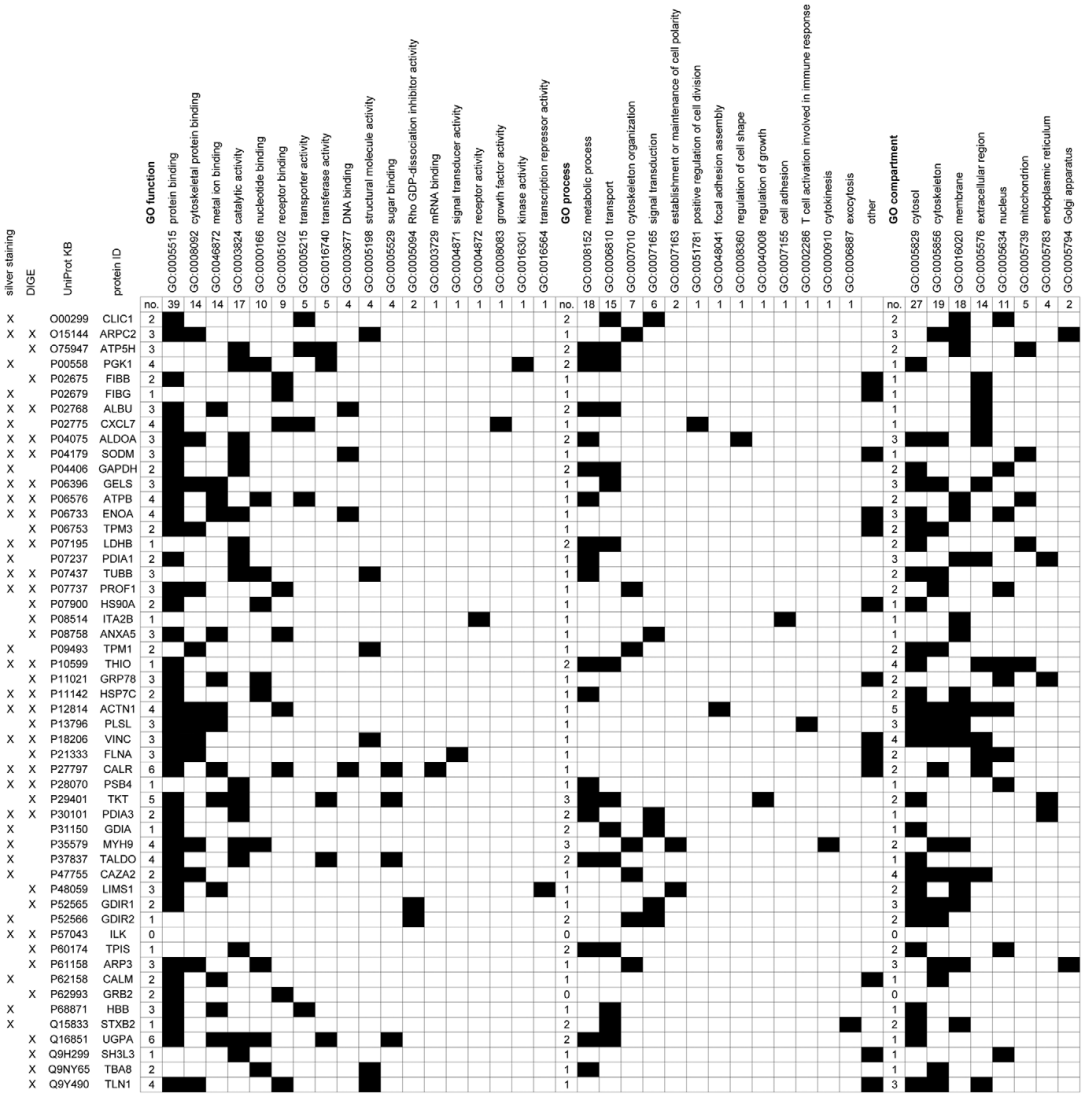
Classification of the differentially expressed proteins according to their cellular function, cellular process and their cellular localization. The graphs display the results following the classification of the subset of differentially expressed proteins represented in single target spots identified via peptide mass fingerprinting according to 18 selected cellular functions (left panel), 13 selected cellular processes (middle panel) and 8 cellular localizations (right panel) along with the respective GO annotation codes. Proteins which could not be assigned to any of these selected processes were classified as “other”. The total number of proteins identified is 52. The 1^st^ and 2^nd^ columns indicate the profiling strategy. The 3^rd^ and 4^th^ columns provide the individual UniProtKB and the protein ID’s. The numbers along the x-axis indicate the number of targets grouped under the given GO annotation code, whereas the numbers on the y-axis the number of shared characteristics or compartments, respectively.

Based on current gene ontology information the 52 differentially expressed proteins represented in single target spots were classified according to their cellular function, the cellular process they are involved in and their cellular localization. The clustering according to the cellular function was restricted to a subset of 18 gene ontology (GO)-annotated functions that might have an impact during T cell activation (Figure 4). Similarly, 13 cellular processes and 8 distinct cellular compartments likely involved in this process were selected. Moreover, the clustering was not restricted to a single feature, therefore the majority of the differentially expressed proteins could be linked to several cellular functions, various processes or different cellular compartments.

The most dominant cellular function is protein binding assigned to 39/52 differentially expressed proteins, followed by 17 proteins exerting catalytic activity, 14 each representing binders of cytoskeletal proteins or metal ions, respectively, 10 binding nucleotides and 9 binding to receptors. Less frequently represented are proteins displaying transporter or transferase activity (5) or proteins binding DNA, with structural molecule activity or binding sugars, (4). Seven of the selected functions, including Rho GDI activity, mRNA binding, signal transducer activity, receptor activity, growth factor activity and transcription repressor activity are only rarely represented (Figure 3). Moreover, several of the identified proteins are linked to multiple functions, in particular CALR, UDP-glucose pyrophosphorylase 2 (UGPA), transketolase (TKL), CXCL7, phosphoglycerate kinase 1 (PGK1), ENOA, transaldolase (TALDO), ACTN1, talin (TLN1) and myosin 9 (MYH9) (Figure 4).

Concerning cellular processes linked to T cell activation 18 out of 52 differentially expressed proteins are associated with metabolic processes, 15 with transport, 7 with cytoskeleton organization and 6 with signal transduction processes. In contrast, proteins involved in the establishment or maintenance of cell polarity, positive regulation of cell division, focal adhesion assembly, regulation of cell shape, regulation of growth, cell adhesion, direct with T cell activation, cytokinesis and exocytosis were rarely detected (Figure 4). Again a number of the identified proteins are linked to several cellular processes, such as TALDO and MYH9 to 3 distinct categories and 13 at least to 2, including CXCL7, GAPDH, LDHB, GDIR2 and others.

With respect to the cellular localization 27/52 of the differentially expressed proteins are assigned to the cytosol, 19 to the cytoskeleton, 18 to the membrane, 14 to the extracellular region and 11 to the nucleus. Less proteins were derived from mitochondria, the endoplasmic reticulum (ER) and the Golgi apparatus, at numbers of 5 to 4 to 2, respectively (Figure 4). ACTN1 can be assigned to 5 compartments, VINC, THIO and F-actin capping protein alpha-2 (CAZA2) to 4 and a panel of 9 proteins, including ARPC2, GELS, ENOA, protein disulfide isomerase A1 (PDIA1), plastin (PLSL), Rho GDI dissociation inhibitor 1 (GDIR1), actin-like protein 3 (ARP3) and TLN1 to 3 distinct compartments (Figure 4).

### Verification of Differentially Expressed Proteins by Flow Cytometry

**Figure 5:**
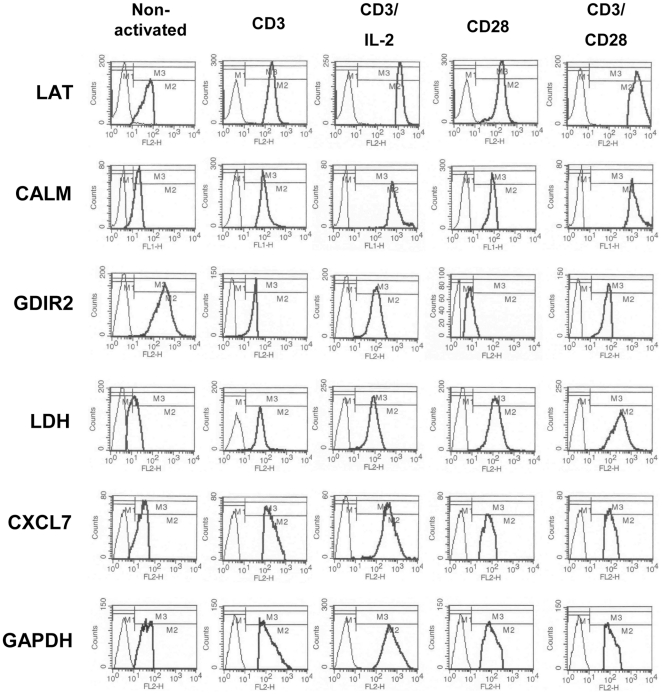
Verification of differentially regulated proteins by flow cytometry. Isolated resting T cells were activated for 48 hrs with the agonistic anti-CD3 antibody OKT3, anti-CD28 antibody 15E8 and IL-2. Incubation with non-activating isotype control mAbs were performed for comparison. T cells were analyzed for the respective proteins by flow cytometry. Solid and dotted histograms represent staining with a specific antibody and an isotype control antibody, respectively.

**Figure 6:**
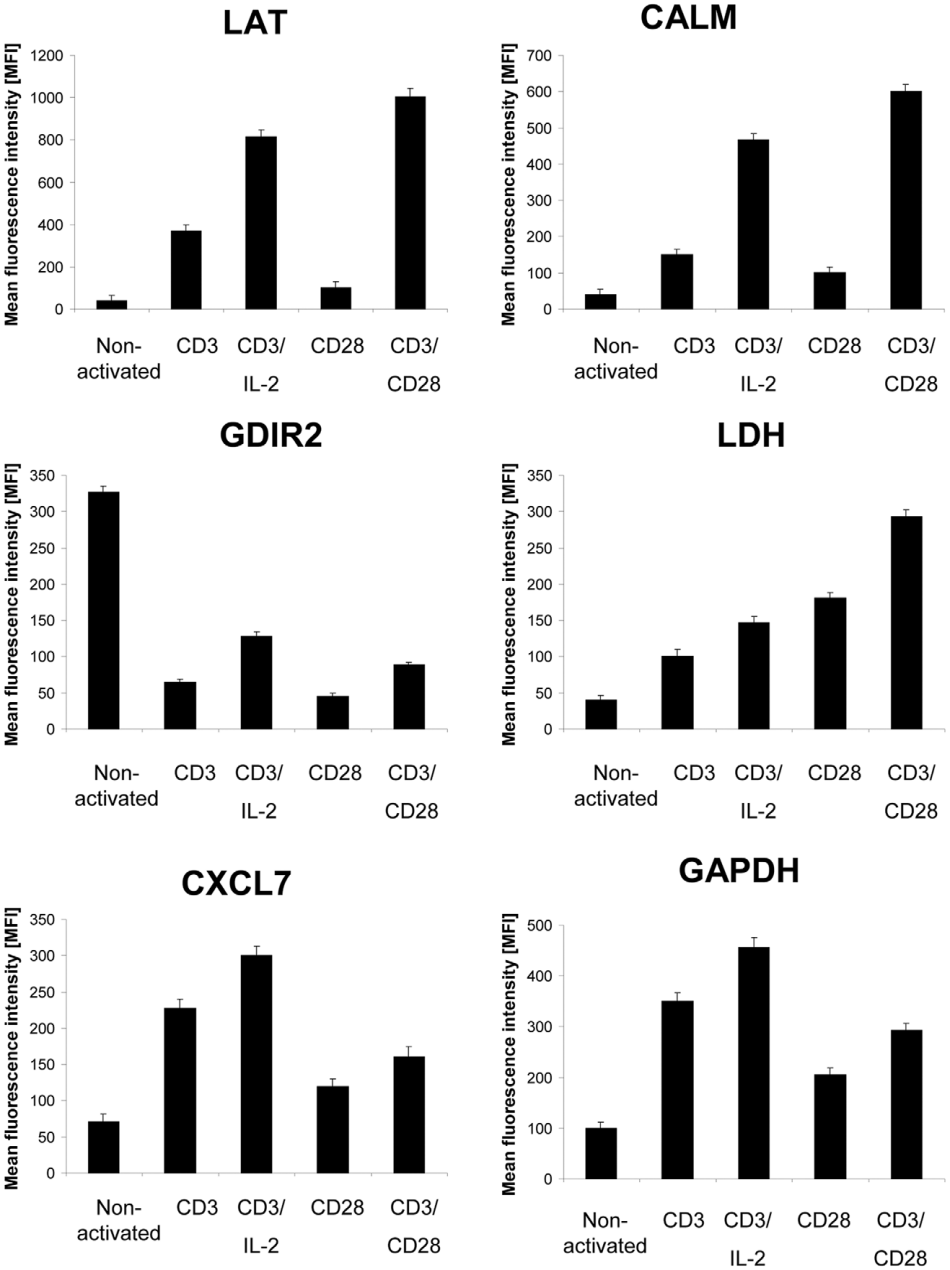
Combined flow cytometric analysis of T cells from five healthy donors. The bar charts represent the mean of mean fluorescence intensity (MFI) ± standard error of mean (SEM) for the indicated target proteins. Statistical analyses were performed using an unpaired t-test.

Since the majority of the differentially expressed proteins in response to the distinct stimulation conditions was involved in metabolic processes, the reorganisation of the cytoskeleton and signal transduction pathways, the expression pattern of representative members of these cellular processess were independently assessed by flow cytometric analyses. The panel of verified proteins is comprised of CALM, a component of the cytoskeleton and regulator of cytokinesis; Rho GDP-dissociation inhibitor 2 (GDIR2), a member of G-protein signaling pathways; CXCL7, which stimulates DNA synthesis, mitosis and glycolysis; and the glycolytic enzymes LDH and GAPDH. Moreover, the expression level of linker for activation of T cells family member 1 (LAT) as an indicator for effective TCR stimulation was also determined by flow cytometry (Figure 5). CD3/CD28 co-stimulation led to the highest LAT expression levels followed by CD3 plus IL2 stimulation whereas stimulation via CD3 or CD28 alone expectedly resulted in low LAT expression. Concomitantly, CALM was highly expressed, which is in accordance to the fact that full T cell activation is calcium dependent and accompanied by a reorganization of the cytoskeleton. In contrast, GDIR2 expression is down-regulated under all costimulatory conditions. Glycolytic enzymes, however, are differentially expressed in response to different stimulation modes. The highest LDH expression levels were detected in CD3/CD28-stimulated T cells, whereas CD3/IL2 or partial activation via CD3 or CD28 led to a less pronounced LDH induction. All costimulatory conditions induced the glycolytic pathway. GAPDH, however, was highly expressed in response to CD3/IL-2 stimulation whereas the other costimulation modes induced lower GAPDH levels. This is also the case for the expression of CXCL7, revealing an expression pattern similar to GAPDH. The observed protein regulation pattern is stable as shown by Figure 6, representing bar graphs for the profiling results of 5 independent biological replicates.

## Discussion

The expression profiling of T cells in response to defined stimuli by gel-based 2-DE using two different protein staining strategies revealed a defined subset of differentially regulated genes. Whereas both protocols revealed roughly the same number of differentially regulated spots there were some differences. DIGE-based profilings revealed more down- and non-regulated spots than silver stainings, although both procedures showed nearly the same numbers of up-regulated proteins (Table 1). The increased number of none regulated spots may likely be attributed to differences in the matching concepts of the imaging software, which for the Delta2D software package (DIGE-based profiling) relies on a 100 percent matching strategy, but for the Proteomweaver software (silver staining-based profiling) on a further distinction between matched and unique spots for the expression profile comparison.

However, the profiling results provide novel insights into the changes occuring in response to distinct activation signals at the protein expression level in T cells, independent of the used staining strategy. Noteworthy the panels of differentially expressed proteins defined by both strategies represent proteins with similar functions, involved in similar processes and frequently represented in more than one cellular compartment (Figure 4). The key celluar processes delineated from the panel of differentially expressed proteins are consistent with known T cell activation-related processes. Thus, the activation of signal transduction pathways, the induction and maintenance of complex metabolic processes, the transport of cellular components into different cellular compartments and along the cytoskeletal backbone, or the reorganization of the cytoskeleton fully support the activation process and its subsequent initiation of cell divisions. The operation of these processes involves 2 to 5 cellular compartments, a feature shared by the majority of the defined differentially expressed proteins (Figure 4).

In general the profiling data resulted in both the description of novel targets, but also the confirmation of previously reported findings. The characterization of the plasma membrane-proximal T cell activation responses in Jurkat T cells led to the identification of a complex protein network that is anchored in the vicinity of activated TCR [38]. Highly enriched in the membrane domains of activated T cells are proteins involved in early signaling events including the tyrosine kinase zeta-chain-associated protein kinase 70 (ZAP-70), which is recruited to CD3 subunits of the TCR in response to the phosphorylation of tyrosine-based activation motifs by kinases (Lck and Fyn). ZAP-70 subsequently phosphorylates LAT leading to the recruitment of the adaptor proteins growth factor receptor-bound protein 2 (GRB2), GRB2-related adaptor protein 2 (GADS) and SLP adaptor and CSK-interacting membrane protein (SLP76) as well as to the recruitment and activation of phospholipase C (PLC) gamma 1 triggering Ca^2+^ flux along with the translocation of the transcription factor nuclear factor of activated T-cells (NFAT) from the cytosol to the nucleus. Furthermore, GRB2 and son of sevenless homolog (SOS) are also involved in CD28 signaling leading to the activation of the mitogen-activated protein kinase (MAPK) pathway and the formation of the activator protein 1 (AP-1) complex resulting in the production of IL-2 [39]. Given that tyrosine kinases Lck and Fyn due to their relative low abundance were merely detectable by immunostainings but not by silver staining [40] might explain their missed detection in the current profiling study. In addition, it has been demonstrated that LAT, a transmembrane protein, fails to enter the isoelectrofocussing gel matrix and thus cannot be detected by 2-DE-based expression profiling [40] thereby virtually also excluding the detection of common T cell activation markers such as as CD25 (IL-2 receptor), CD69 (activation inducer molecule), CD71 (transferrin receptor) or HLA-DR (human lymphocyte antigen class II cell surface receptor) for the same reason. However, it provided the rational for the validation of LAT by flow cytometry as an early activation marker. Moreover, the PLC gamma 1-induced triggering of the Ca2+ flux was validated by monitoring the expression pattern of CALM in response to the various stimulation conditions (Figures 5 and 6), taking further into account that it had been previously defined as modified in response to CD28-mediated costimulation [36]. Furthermore, the molecular chaperone HSP7C was identified in the context of T cell activation as a lipid raft-associated protein [40] (Table S2), presumably supporting MAPK signaling, antigen processing and presentation as well as endocytosis.

The expression of CXCL7, up-regulated in CD3 and in CD28 partially activated T cells, is in line with previous studies on the synthesis of leukocyte-derived growth factor in T lymphocytes [41]. The protein is thought to be required for supplying the secreted cleavage product CXCL7 in an autocrine fashion. CXCL7 induces chemotaxis, supports the clustering of cells as frequently observed for expanding T cells, and is also involved in the induction of glucose uptake by inducing glucose transporter 1 (GLUT1) expression [42], thus supplying the activated cell with energy resources. In addition, CXCL7 acts as a positive regulator of cell growth displaying characteristics of a mitogene [41]. In line with increased glucose uptake several enzymes associated with the energy metabolism were found to be upregulated (Table S2). These include in particular components of the glycolysis and pentose phosphate pathways, such as ALDOA, ENOA, GAPDH, LDHB and TKT, which previously had been identified as differentially expressed during T cell activation [38], [43], [44]. In addition PGK1, triosephosphate isomerase (TPIS) and TALDO, were also defined as differentially expressed in response to distinct costimulatory signals (Table S2). These data are in line with earlier profiling results [36] thereby providing the rational for the selection of GAPDH and LDH as representative validation targets (Figures 5 and 6). In this context it is further noteworthy that some isoforms of GAPDH, ALDOA and ENOA were also found to be down-regulated in response to co-stimulatory signals, although the nature for any of these isoforms has still to be defined.

The establishment of focal adhesions in response to TCR activation as delineated from the current profiling results is in line with the data reported by Nguyen and co-authors [43]. ACTN1 and TLN1 have been demonstrated to associate Ca^2+^-dependent with integrin alpha-L (LFA-1), which under physiological conditions binds to intercellular adhesion molecule 1 (ICAM-1) on APC, thereby initiating cell cell contact. ACTN1 was even detected in several independent spots, including spots displaying inverse regulation modes indicating that this protein might undergo a series of post-translational modifications (PTM) in response to the activating stimuli. Along with filamin-A (FLNA), VINC (Table S2) and ACTB (Tables S1 and S3) these differentially expressed proteins cover almost the entire focal adhesion pathway.

In line with that, proteins involved in the organization of the cytoskeleton are up-regulated, alike the tropomyosin 1 alpha chain (TPM1) (Table S2), which is involved in the movement of cellular components and the regulation of cell migration or myosin-9 (MYH9), which is involved in important cellular processes like the establishment of T cell polarity, cell morphogenesis involved in differentiation as well as in the organization of the mitotic spindle. Concomitantly, GELS that is linked to the disassembly of cellular components and the depolymerization of actin filaments was found to be down-regulated (Table S2). Furthermore, the up-regulated syntaxin-binding protein 2 (STXB2), a protein linked to vesicle-mediated transport, supports the finding that a considerable number of the differentially expressed proteins seem to be involved in modulating the extracellular compartment.

Further evidence that T cell activation is associated with dynamic changes of the actin cytoskeleton comes from a recent report of Lin and co-workers [44] analyzing the composition of lipid raft proteins in response to chemokine stimulation in Jurkat cells thereby demonstrating that the molecular chaperone heat shock protein HSP 90-alpha (HS90A) not only acts as scaffolding site in lipid rafts, but also appears to be involved in stimulating/mediating Ca^2+^ flux and in the regulation of the structure of actin filaments assembled by neuronal Wiskott-Aldrich Syndrome protein (N-WASP) and the Arp2/3 complex thereby activating the small GTPases Rac and Rho, which can both be linked to mediate cell migration. The observed up-regulation of HS90A in response to costimulatory signals (Table S2) suggests a similar role for HS90A in the context of this type of T cell activation. Furthermore, other proteins involved in the rearrangement of the actin cytoskeleton, like CALM, ARPC2, CAZA1, PROF1 and PLSL are actively recruited into lipid rafts [44] and exert a similar regulation pattern in response to activation of T cells by costimulatory signals. The Ca^2+^-mediated up-regulation of CALM and PLSL (Table S2) in combination with the down-regulation of PROF1 strongly support the reorganization of the actin cytoskeleton and the favoring of actin polymerization, dispite the fact that ARPC2 was found to be down-regulated, which however might be compensated by the up-regulation of CAZA2 (Table S2). Moreover, these results are also in line with the phosphoproteomic profiling data presented by Nguyen and co-workers [43] revealing that CALM, GDIR2 and LAT are differentially expressed during T cell activation, leading to the selection of these representative proteins to the group of validated targets (Figures 5 and 6).

Concerning the expression pattern of proteins associated with signal transduction pathways, the up-regulation of ILK, which together with LIM and senescent cell antigen-like containing domain protein 1 (LIMS1), is up-regulated in CD28-stimulated T cells acts as a receptor proximal protein kinase and regulates integrin-mediated signal pathways, such as the formation of focal adhesions. However, another isoform of ILK is consistently down-regulated. PDIA3 has isomerase activity, interferes with signaling pathways, is a potential positive regulator of apoptosis and is up-regulated under certain costimulation conditions. Moreover, the down-regulation of GDIR2, a regulator of small GTPase-mediated signal transduction pathways and a negative regulator of cell adhesion is in accordance to the T cell activation process as is the down-regulation of the functionally related Rab GDP dissociation inhibitor alpha (GDIA) that is reacting to calcium influx (Table S2).

Recent profiling data addressing the altered protein expression pattern in T cells following phytohemagglutinin (PHA) stimulation defined a panel of differentially expressed proteins [37]. Based on the current profiling approximately 50% thereof are also modulated in response to costimulation-mediated T cell activation. The overlap amongst the groups of down-regulated proteins is higher (60%) than for the group of up-regulated proteins (35%). For instance, ACTN1, GAPDH, HSP7C, PDIA3, SODM, TBB5 and VINC, within the groups of down-regulated proteins or PGK1 and ALDOA representing examples for up-regulated proteins. In addition, ACTN1, ALDOA, GAPDH, VINC and TLN1 were also represented by several independent spots, likely due to undergoing PTM (Table S1, Table S2 and Table S3).

Unexpectedly, none of the up-regulated isoforms of GAPDH reached the required threshold levels (factor: >2 silver staining; >1.5, DIGE). However, taking into account that GAPDH is prone to undergo massive PTM (Figure 2) it is likely that not all of its variants were identified. Though at least the PTM-independent flow cytometric stainings (Figure 5 and 6) confirm the induction of GAPDH at the total protein expression level in response to the different stimulation.

Furthermore, the data presented by Sheng and Wang [37] suggest that GAPDH might play a role as transcriptional regulator during T cell activation since it is co-immunoprecipitated in ChiP assays. In addition to its central function in glycolysis, there is increasing evidence, that GAPDH is also linked to DNA repair, tRNA export, membrane fusion and transport, cytoskeletal dynamics as well as cell death [45]. The diverse functions of GAPDH are dependent on its grade of oligomerization, posttranslational modifications and its cellular localization. Under normoxic conditions GAPDH is predominantly expressed in the cytosol, functioning as a housekeeping protein in the glycolysis by forming a tetramer. Under oxidative stress GAPDH can undergo S-thiolyation, leading to a metabolic shift towards the pentose phosphate pathway. In addition the redox-sensitive cysteine residue of GAPDH binds to inositol 1,4,5-trisphosphate receptors in close proximity to calcium channels of the ER membrane, thereby regulating Ca2+-mediated signaling [46]. Thus, the up-regulation of CALM in response to CD3/CD28 and CD3/IL2 mediated T cell activation may be linked to the increased GAPDH expression levels.

Taking further into account that GAPDH can also interact with tubulins and actins [47], [48] the differential expression of various tubulins (tubulin alpha-1A chain (TBA1A), tubulin alpha-1B chain (TBA1B), tubulin alpha-1C chain (TBA1C), tubulin alpha-4A chain (TBA4A), tubulin alpha-8 chain (TBA8) and TBB5) as well as of isoforms of ACTB and/or ACTG are plausible (Table S2). The binding of GAPDH to microtubules leads to a reduction of its glycolytic activity due to the dissociation of its metabolic active tetrameric structure into inactive monomers [49]. In addition, enzymatically active GAPDH can be transported within the cell via the microtubule treadmill. In particular phosphorylated isoforms of GAPDH are involved in mediating vesicular trafficking between cellular compartments, such as between the ER and the Golgi apparatus, by acting as an adaptor or scaffolding protein [50]. This role of GAPDH may be relevant during T cell activation given the fact that differentially expressed proteins including signaling molecules like CXCL7 or PDIA1 as well as structural molecules, like ACTN1, GELS, VINC, TLN1 along with the anti-oxidants THIO next to several others can reach the extracellular region under physiological conditions (Figure 4). Although under stress conditions GAPDH is predominantly S-nitrosylated, interacts with Siah, an E3 ubiquitin-protein ligase [51] and is then translocated to the nucleus leading to cellular dysfunction and cell death, it can also be translocated into the nucleus without induction of cell death likely post undergoing O-GlcNAcylation in the cytosol [52] via an as yet undisclosed mechanism. Subsequently GAPDH can bind to protein SET, increasing mitosis by acceleration of the cell cycle, due to higher cyclin B-cdk1 activity [53]. Taken together, posttranslational modifications of GAPDH reflect the various activation-associated alterations in transport, metabolism and signaling of activated T cells.

Nyman and colleagues [54] reported a 2-DE-based map of CD3/CD28-stimulated CD4^+^ helper cells providing the identification of 91 spots representing 72 proteins. Fourteen of the identified proteins were represented by more than one spot implying posttranslational modifications; however, none of the identified proteins was linked to cellular processes. However, based on the current report, 15 of these proteins can now be associated with cellular processes linked to T cell activation, namely mitochondrial stress-70 protein 70 (GRP75), HSP7C, 78 kDa glucose-regulated protein (GRP78), PDIA3, ENOA, ATPD, PGK1, ACTB, TPIS, GRB2, GDIR1, GDIR2, ATP synthase subunit d (ATP5H) and PROF1.

From the overall 30 proteins that previously had also been identified as differentially expressed in plasma membrane proximal domains during T cell activation [38] 8/11 defined as up-regulated maintained their regulation mode, including the metabolic enzymes ENOA and LDHB, the cytoskeleton components TBB5, MYH9, PROF1, TLN1 and PLSL as well as the stress protein HSP90A (Table S2). In contrast, GAPDH and the signaling components GBR2 and GDIR2 defined as up-regulated in membrane-proximal domains upon T cell activation were rather defined as down-regulated in this 2-DE-based profiling report (Table S2), which at least for GDIR2 was confirmed (Figures 5 and 6). Nonetheless, this still does not rule out its enrichment in receptor-proximal membrane domains. For some of these targets, like PROF1, TBB5, ENOA, TLN1 and HSP90A also counter-regulated spots were detected. However, since these proteins can undergo PTMs, this is not unexpected and represents an advantage of the gel-based versus the corresponding peptide-based SILAC approach.

Within the group of targets that was previously classified as down-regulated, 8/13 of the shared proteins maintained their regulation mode, including the metabolic enzymes ATP5B, ATP5H, PDIA1 and PDIA3, the cytoskeleton components ARPC2 and FLNA as well as ALBU and CALR (Table S2). A partial counterregulation was detected for ATPB and PDIA3, whereas the cytoskeleton components ARP3, and CAZA2, the signaling component CALM next to GRP78 and STXB2 exerted a general counterregulation. However, the inverse regulation identified for CALM (Table S2) is again supported by the validation data (Figures 5 and 6).

Moreover, for the metabolic enzymes ALDOA, PGK1 and SODM, the cytoskeleton components ACTN1 and tropomyosin alpha-3 chain (TPM3) as well as for hemoglobin subunit beta (HBB) the current profiling now defines the respective regulation pattern (Table S2). The role of ALDOA appears to depend on certain PTM since both up-regulated and down-regulated ALDOA spots could be detected, in particular in partially activated T cells. An inverse regulation pattern were also defined for ACTN1, which seems to be up-regulated in partially stimulated, but down-regulated in fully activated T cells. For PGK1 the up-regulation is in line with the concept that stimulated T cells require higher energy levels. In contrast, SODM is down-regulated under all stimulation conditions, in line with the report by Kronfeld and colleagues [36]. TPM3, previously defined as phosphorylated in response to costimulation [36] is down-regulated, in at least CD3 + IL2-stimulated T cells, whereas HBB defined as up-regulated in CD28-stimulated T cells seems to be rather inversely regulated based on the current profiling data. However, the function of both proteins during T cell activation still needs to be defined.

This is also the case for a subset of differentially expressed proteins that has not yet been linked to the costimulation-mediated T cell activation process..From the group of metabolic enzymes UGPA is likely involved in the metabolism of sugars, whereas TPIS, TALDO and TKT represent components of the glycolysis and pentose phosphate pathways, respectively (Table S2). Analogous to SODM THIO is involved in counteracting the increase of reactive oxygen species during T cell activation as previously described for murine T cells by Michalek and coworkers [55]. The proteasomal subunit PSB4, previously also defined as phosphorylated in response to costimulation [36] is likely contributing to the ubiquitin-mediated proteolysis of cell surface and signaling molecules in response to the activation of the TCR signaling pathway [38]. From the group of components of the cytoskeleton, VINC is bridging the gap in the focal adhesion pathway between ACTN1, FLNA and TLN1 and the initiation of actin polymerization, while GELS and TBA8 are involved in the reorganization of the actin cytoskeleton. The signaling components GDIR1 and GDIR2 are both considered as regulators of small GTPase- and Rho protein-mediated signal transduction processes, whereas the functions of the down-regulated targets annexin A 5 (ANXA5), chloride intracellular channel protein 1 (CLIC1) and GDIA (Table S2) in the process of T cell activation still have to be elucidated.

This also holds up for proteins that could not be categorized into the 3 main groups (Table S2), in particular for the secreted proteins fibrinogen beta chain (FIBB), fibrinogen gamma chain FIBG and the membrane–associated integrin alpha IIb (ITA2B), wheras for SH3 domain-binding glutamic acid-rich-like protein 3 (SH3L3) a function towards (re-)establishing redox homeostasis seems likely.

Taken together the profiling data presented in this report provide novel insights into the complex alterations of the proteome during T cell activation, in particular in regard to alterations in the metabolism, the reorganization of the cytoskeleton and the initiation/modulation of signaling pathways. Thus, the profiling results increase the knowledge with respect to the network of underlying biological processes associated with the activation of T cells beyond the individual signal transduction pathways.

## Materials and Methods

### Isolation of T Cells

CD3^+^ T cells were isolated from the peripheral blood of healthy human donors. In brief, mononuclear cells (PBMC) were isolated by Ficoll-Paque gradient centrifugation (Amersham Biosciences, Amersham, UK), CD3^+^ T cells purified by negative selection with the “MACS human pan T cell isolation kit” using the AutoMACS Pro separator (Miltenyi Biotech, Bergisch Gladbach, Germany). T cell purity was routinely >98% as determined by flow cytometry (FACS Canto, BD Biosciences, Heidelberg, Germany). Cells were cultured in X-Vivo15 medium containing 10% (v/v) fetal calf serum (FCS), 1% (v/v) Penicillin/Streptomycin (100 X; 10.000 U/ml/10 mg/ml) and 2 mM glutamine (all purchased from Sigma Aldrich, Deisenhofen, Germany).

### T Cell Activation

Polysorb culture plates (Nunc, Roskilde, Denmark) were coated with the anti-CD3 antibody OKT3 (Natutec, Frankfurt/Main, Germany, 10 µg/ml coating concentration), the anti-CD28 antibody 15E8 (CLB, Amsterdam, Netherlands, 10 µg/ml), both OKT3 and 15E8 antibodies (each 10 µg/ml), and OKT3 antibody (10 µg/ml) and IL-2 (Endogen, Woburn, MA, USA, 1000 U/ml. Plates coated with an isotype matched IgG antibody (10 µg/ml) served as controls. Isolated CD3^+^ T cells were cultured in coated plates in complete X-Vivo15 medium (Lonza, Cologne, Germany) for 48 hrs.

### Two-dimensional Gel Electrophoresis (2-DE) and Difference Gel Electrophoresis (DIGE)

Isolated CD3^+^ T cells after antibody stimulation or incubation with an isotype-matiched IgG antibody as control were initially subjected to 2-DE-based expression profilings by loading 150 µg protein per sample. Proteins were visualized by silver stainings as previously described [56]. In addition, quantitave profilings were performed by applying an DIGE approach based on a minimal labeling strategy according to the manufacturer’s instruction (Amersham Biosciences, Freiburg, Germany). For minimal labelings, 15 µg protein representing a mixture of equal amounts of total lysates derived from stimulated (OKT3, 15E8, OKT3 plus IL2, OKT3 and 15E8) and non-stimulated (IgG isotype) CD3^+^ T cells were labeled with CyDye DIGE fluor 2 as internal protein standard (IPS). In parallel 15 µg total protein lysates from either stimulated or non-stimulated CD3^+^ T cells were separately labeled with CyDye DIGE fluors 3 and 5, respectively. Each labeling reaction was performed in a total volume of 20 µl DIGE lysis buffer (30 mM Tris, 7 M urea (Applichem, Darmstadt, Germany), 2 M thiourea (Sigma, Deisenhofen, Germany) 4% (w/v) CHAPS (Applichem), adjusted to pH 8.5) after adding 100 pmol CyDye (Amersham Biosciences) dissolved in 1 µl dimethylformamide for 30 minutes on ice. The labeling reaction was stopped by adding 1 µl 10 mM lysine (Merck Biosciences GmbH, Schwalbach, Germany) followed by 10 minutes on ice. Isoelectric focusing using Immobiline DryStrips pH 3–10 NL (Amersham Biosciences) and second dimension SDS separation were performed as previously described [56]. Gels were washed three times in destilled water and thereafter scanned using a Fuji FLA 5100 fluorescence scanner (Fuji Photo Film GmbH, Düsseldorf, Germany) equipped with 3 lasers. Cy2 images were acquired with a blue laser (473 nm) in combination with a LPB filter (510 nm), whereas Cy3 and Cy5 with green (532 nm) and red lasers (635 nm) in combination with LPG (575 nm) and LPR (665) filters, respectively. For all scannings the voltage applied to the photomultiplier tubes (PMT) was set to 50 V below the saturation level of the most abundant protein spot within the given gel (ranging from 600–800 V). All gel images were recorded at a resolution of 100 µm.

### Image Analysis

Quantitative image analyses of 2-DE gels were performed using either the Proteomweaver (Version 4.0, Bio-Rad GmbH, Munich, Germany) or for DIGE gels the Delta2D (Version 4.2, Decodon GmbH, Greifswald, Germany, DIGE) software packages according to the manufacturer’s guidelines. Spots displaying upon stimulation either a relative up- or down-regulation ratio of factor ≥2.0 (silver staining) or >1.5 (DIGE) compared to the respective control were classified as differentially expressed and selected for subsequent micro-analytical characterization.

### Mass Spectrometry and Data Mining

Preparative gels were loaded with 750 µg total protein of cell lysates, the spots of interest were subsequently digested in the respective gel in situ and subjected to mass spectrometry using the matrix-assisted laser-desorption/ionization time of flight (MALDI-TOF) instrument Voyager DE™ PRO (Applied Biosystems, Forster City, CA, USA) as previously described [57]. However, due to the very limited sample material available, preparative gels were run with samples representing unfractionated Ficoll-isolated T cells obtained from healthy donors. Therefore, the analysis was only restricted to those spots, which could be matched between the profiles of the crude and highly purified T cell preparations. Database searches were performed using the MASCOT software package (Matrix Science, London, UK). Data obtained from free accessible data banks were analyzed with an in-house web-based tool and linked to a mySQL database (db) as recently described [58]. The data sets of the identified genes and proteins were linked to the Swiss-Prot identities (UniProt Knowledgebase Release 2011_05).

### Flow Cytometry

T cells were washed twice in 2 ml FACSFlow (BD Biosciences, Heidelberg, Germany) containing 0.1% (w/v) bovine serum albumin (Sigma Aldrich) and resuspended to a concentration of 20×10^6^ per ml. Cells were fixed and permeabilized using the “INTRASure kit” (BD Biosciences). Washed cells were incubated with 10 µl each of titrated antibody directed against LAT (BD Biosciences), CALM (Abcam, Oxford, UK), GAPDH (Cell Signaling, Frankfurt/Main, Germany), LDH (Abcam), GDIR2 (Lifespan Biosciences, Seattle, USA) or CXCL-7 (Santa Cruz Biotech, Heidelberg, Germany), respectively, for 20 min at 4°C in the dark, washed and 1 µg/tube of fluorochrome-conjugated secondary antibody was added for 20 min at 4°C. As control an isotype-matched IgG was used as primary antibody. Cells were fixed with 1% (w/v) PFA (Sigma Aldrich) and analysed, on a “FACSCanto III” flow cytometer. Data were analysed using FACSDiva software (BD Biosciences) with a minimum of 10,000 events. Live cells were recorded prior the fixation step utilizing 7-AAD.

## Supporting Information

Table S1 List of differentially expressed proteins defined by silver staining (regulation factor >2).
(PDF)

Table S2 List of differentially expressed proteins classified according to their cellular function.
(PDF)

Table S3 List of differentially expressed proteins (DIGE minimal labeling, regulation factor >1.5).
(PDF)

Table S4 List of identified proteins with respective accession numbers, predicted and experimental molecular weight (MW) as well as sequence coverage and score obtained by Mascot database search.
(PDF)
