# Supplementary material for: A Proteomic View at T Cell Costimulation
Source: PLoS One. 2012 Apr 23;7(4):e32994. doi: 10.1371/journal.pone.0032994 (PMC3335147; doi:10.1371/journal.pone.0032994)
Supplement: Table S1 — List of differentially expressed proteins defined by silver staining (regulation factor >2). (PDF) [file pone.0032994.s001.pdf]

Table S1: List of differentially expressed proteins defined by silver staining (regulation factor > 2)

A) up-regulated proteins

	CD3/PBS	CD28/PBS	CD3 + CD28/PBS
shared single target spots	CAZA2, P47755		CAZA2, P47755
	CXCL7, P02775	CXCL7, P02775	
	TPM1, P09493		TPM1, P09493
single target spots	ACTN1, P12814	CALM, P62158	MYH9, P35579
	ALDOA, P04075	HBB, P68871	STXB2, Q15833
	ILK, P57043	PGK1, P00558	THIO, P10599
	PROF1, P07737		
	VINC, P18206		
multi ple target spots	ACTB, P60709/ACTG, P63261		ACTB, P60709/ACTG, P63261
	ACTB, P60709/ACTG, P63261		ACTN1, P12814/VINC, P18206

B) down-regulated proteins

	CD3/PBS	CD28/PBS	CD3 + CD28/PBS
shared single target spots	GELS, P06396	GELS, P06396	GELS, P06396
single target spots	ALBU, P02768	ALDOA P04075	ACTN1, P12814
	ARPC2, O15144	ATPB, P06576	FIBG, P02679
	CALR, P27797	CLIC1, O00299	PSB4, P28070
	PDIA1, P07237	ENOA, P06733	PDIA3, P30101
	TALDO, P37837	ENOA, P06733	SODM, P04179
	TBB5, P07437	GAPDH, P04406	
	VINC, P18206	GAPDH, P04406	
		HSP7C, P11142	
		LDHB, P07195	
		GDIA, P31150	
		GDIR2, P52566	
multiple target spots		ACTB, P60709/ ACTG, P63261	TBA1B, P68363/TBA1C, Q9BQE3
		ACTB, P60709/ ACTG, P63261	
		TPM2, P07951/K1C10, P13645	
