# Supplementary material for: A Proteomic View at T Cell Costimulation
Source: PLoS One. 2012 Apr 23;7(4):e32994. doi: 10.1371/journal.pone.0032994 (PMC3335147; doi:10.1371/journal.pone.0032994)
Supplement: Table S2 — List of differentially expressed proteins classified according to their cellular function. (PDF) [file pone.0032994.s002.pdf]

Table S2: List of differentially expressed proteins classified according to their cellular function

A) up-regulated proteins

stimul. mode	CD3/PBS	CD3 + Il-2/PBS	CD28/PBS	CD3 + CD28/PBS
**metabolic process**				
	ALDOA, P04075 *			
		ATP5B, P06576 #		
	ENOA, P06733 #	ENOA, P06733 #		
				HSP7C, P11142 #
			LDHB, P07195 #	
		PDIA3, P30101 #	PDIA3, P30101 #	
			PGK1, P00558*	
				THIO, P10599 *
	TPIS, P60174 #		TPIS, P60174 #	
			UGPA, Q16851 #	UGPA, Q16851 #
**cytoskeleton organization**				
	ACTN1, P12814 *	ACTN1 P12814 #	ACTN1 P12814 #	
			ARP3, P61158 #	
	CAZA2, P47755 *			CAZA2, P47755 *
				GELS, P06396 #
				MYH9, P35579 *
	PROF1, P07737 *			
	TBB5, P07437 #	TBB5, P07437 #		
				TLN1, Q9Y490 #
	TPM1, P09493 *			TPM1, P09493 *
		PLSL, P13796 #	PLSL, P13796 #	PLSL, P13796 #
	VINC, P18206 *	VINC, P18206 #		
**signal transduction**				
			CALM, P62158 *	
	GDIR1, P52565 #			
				HSP7C, P11142 #
	ILK, P57043 *			
			LIMS1, P48059 #	
		PDIA3, P30101 #	PDIA3, P30101 #	
**others**				
	CALR, P27797 #	CALR, P27797 #		
	CXCL7, P02775 *		CXCL7, P02775 *	

	FIBB, P02675 #		
GRP78, P11021 #	GRP78, P11021 #		
		HBB, P68871 #	
HS90A, P07900 #	HS90A, P07900 #		
	ITA2B, P08514 #		
			STXB2, Q15833 *

B) down-regulated proteins

stimul. mode	CD3/PBS	CD3 + Il-2/PBS	CD28/PBS	CD3 + CD28/PBS
	**metabolic process**			
	ALBU, P02768 ‘	ALBU, P02768 #		
	ALDOA, P04075 #	ALDOA, P04075 #	ALDOA, P04075 ‘	
			ATPB, P06576 *	
		ATPH5, O75947 #		
			ENOA, P06733 *	
			GAPDH, P04406 ’	
	HSP7C, P11142 #	HSP7C, P11142 #	HSP7C, P11142 ’	
			LDHB, P07195 *	
	PDIA1, P7237 *			
				PDIA3, P30101 *
		PSB4, P28070 #		PSB4, P28070 *
	SODM, P04179 #	SODM, P04179 #	SODM, P04179 #	SODM, P04179 *
	TALDO, P37837 *			
		THIO, P10599 #		
				TKT, P29401 #
	**cytoskeleton organization**			
				ACTN1, P12814 *
	ARPC2, O15144 *	ARPC2, O15144 #		
		FLNA, P21333 #		
	GELS, P06396 ‘	GELS, P06396 #	GELS, P06396 ’	GELS, P06396 *
		PROF1, P07737 #	PROF1, P07737 #	PROF1, P07737 #
	TBA8, Q9NY65 #	TBA8, Q9NY65 #	TBA8, Q9NY65 #	TBA8, Q9NY65 #
	TBB5, P07437 *	TBB5, P07437 #		TBB5, P07437 #
	TLN1, Q9Y490 #	TLN1, Q9Y490 #	TLN1, Q9Y490 #	
		TPM3, P06753 #		
	VINC, P18206 ‘	VINC, P18206 #	VINC, P18206 #	VINC, P18206 #

**signal transduction**			
	ANXA5, P08758 #		
		CLIC1, O00299 *	
		GDIA, P31150 *	
		GDIR2, P52566 *	
		GRB2, P62993 #	
HSP7C, P11142 #	HSP7C, P11142 #	HSP7C, P11142 ’	
ILK, P57043 #	ILK, P57043 #	ILK, P57043 #	ILK, P57043 #
			PDIA3, P30101 *
**others**			
CALR, P27797 *			
		CXCL7, P02775 #	
	FIBB, P02675 #		FIBB, P02675 #
			FIBG, P02679 *
			HS90A, P07900 #
		SH3L3, Q9H299 #	

*, # and ‘ indicate targets either defined based on silver, DIGE or both stainings. The group of others is comprised of targets assigned to functions other than the 3 main categories.
