# Supplementary material for: A Proteomic View at T Cell Costimulation
Source: PLoS One. 2012 Apr 23;7(4):e32994. doi: 10.1371/journal.pone.0032994 (PMC3335147; doi:10.1371/journal.pone.0032994)
Supplement: Table S3 — List of differentially expressed proteins (DIGE minimal labeling, regulation factor >1.5). (PDF) [file pone.0032994.s003.pdf]

Table S3: List of differentially expressed proteins (DIGE minimal labeling, regulation factor > 1.5)

A) up-regulated proteins

	CD3/PBS	CD3 + Il-2/PBS	CD28/PBS	CD3 + CD28/PBS
shared single target spots	CALR, P27797	CALR, P27797		
	ENOA, P06733	ENOA, P06733		
	GRP78, P11021	GRP78, P11021		
	HS90A, P07900	HS90A, P07900		
	TBB5, P07437	TBB5, P07437		
	TPIS, P60174		TPIS, P60174	
		ACTN1 P12814	ACTN1 P12814	
		PLSL, P13796	PLSL, P13796	PLSL, P13796
		PDIA3, P30101	PDIA3, P30101	
		PDIA3, P30101	PDIA3, P30101	
			UGPA, Q16851	UGPA, Q16851
single target spots	GDIR1, P52565	ATPB, P06396	ARP3, P61158	GELS, P06396
		FIBB, P02675	LDHB, P07195	HSP7C, P11142
		ITA2B, P08514	LIMS1, P48059	TLN1, Q9Y490
		VINC, P18206		
multiple target spots	ACTB, P60709/ ACTG, P63261	ACTB, P60709/ ACTG, P63261		ACTB, P60709/ ACTG, P63261
	ACTB, P60709/ ACTG, P63261	ACTB, P60709/ ACTG, P63261		
	ACTN1, P12814/VIME, P08670			ACTN1, P12814/VIME, P08670
	TBA4A, P68366/TBA1A, Q71U36	TBA4A, P68366/TBA1A, Q71U36	TBA4A, P68366/TBA1A, Q71U36	TBA4A, P68366/TBA1A, Q71U36
		TBA4A, P68366/VIME, P08670		TBA4A, P68366/VIME, P08670
		FIBB, P02675/COR1A, P31146	FIBB, P02675/ COR1A, P31146	
			CRCM, P23508/CALX, P27824	CRCM, P23508/CALX, P27824
	TPM2, P07951/TPM4, P67936/ K1C10, P13645	ACTB, P60709/ ACTG, P63261		ACTN1, P12814/K1C10, P13645
				GRP75, P38646/ANXA6, P08133

B) down-regulated proteins

	CD3/PBS	CD3 + Il-2/PBS	CD28/PBS	CD3 + CD28/PBS
shared single protein spots	ALBU, P02768	ALBU, P02768		
	ALDOA, P04075	ALDOA, P04075	ALDOA, P04075	
		ALDOA, P04075	ALDOA, P04075	
	GELS, P06396	GELS, P06396	GELS, P06396	
	HSP7C, P11142	HSP7C, P11142		
	HSP7C, P11142	HSP7C, P11142		
	ILK, P57043	ILK, P57043	ILK, P57043	ILK, P57043
	SODM, P04179	SODM, P04179	SODM, P04179	
	TBA8, Q9NY65	TBA8, Q9NY65	TBA8, Q9NY65	TBA8, Q9NY65
	TLN1, Q9Y490	TLN1, Q9Y490	TLN1, Q9Y490	
	VINC, P18206	VINC, P18206	VINC, P18206	VINC, P18206
	VINC, P18206	VINC, P18206	VINC, P18206	
		PROF1, P07737	PROF1, P07737	PROF1, P07737
		TBB5, P07437		TBB5, P07437
single protein spots	VINC, P18206	ANXA5, P08758	CXCL7, P02775	FIBB, P02675
		ARPC2, O15144	GAPDH, P04406	HS90A, P07900
		ATP5H, O75947	GRB2, P62993	TKT, P29401
		FIBB, P02675	HSP7C, P11142	
		FLNA, P21333	SH3L3, Q9H299	
		PSB4, P28070		
		THIO, P10599		
		TPM3, P06753		
multiple target spots	ACTB, P60709/ ACTG, P63261	ACTB, P60709/ ACTG, P63261	ACTB, P60709/ ACTG, P63261	
	ACTN1, P12814/K1C10, P13645	ACTN1, P12814/K1C10, P13645	ACTB, P60709/ ACTG, P63261	
	FLNA, P21333/VINC, P18206	FLNA, P21333/VINC, P18206	FLNA, P21333/VINC, P18206	FLNA, P21333/VINC, P18206
	GRP75, P38646/ANXA6, P08133	GRP75, P38646/ANXA6, P08133	GRP75, P38646/ANXA6, P08133	FLNA, P21333/GELS, P06396
	TCPD, P50991/K1C10, P13645	TCPD, P50991/K1C10, P13645	TCPD, P50991/K1C10, P13645	TCPD, P50991/K1C10, P13645
		ACTN1, P12814/FLNA, P21333/HS90A, P07900	ACTN1, P12814/FLNA, P21333/HS90A, P07900	ACTN1, P12814/FLNA, P21333/HS90A, P07900
