# Supplementary material for: A Proteomic View at T Cell Costimulation
Source: PLoS One. 2012 Apr 23;7(4):e32994. doi: 10.1371/journal.pone.0032994 (PMC3335147; doi:10.1371/journal.pone.0032994)
Supplement: Table S4 — List of identified proteins with respective accession numbers, predicted and experimental molecular weight (MW) as well as sequence coverage and score obtained by Mascot database search. (PDF) [file pone.0032994.s004.pdf]

Table S4: List of identified proteins with respective accession numbers, predicted and experimental molecular weight (MW) as well as sequence coverage and score obtained by Mascot database search.

protein ID	protein name	UniProtKB ID	MW predicted	MW 2DE	seq. cov.	score
CLIC1	chloride intracellular channel protein 1	O00299	27117	30000	37%	64
ARPC2	actin-related protein 2/3 complex subunit 2	O15144	34426	32000	40%	98
ATP5H	ATP synthase D chain	O75947	18405	23000	65%	90
PGK1	phosphoglycerate kinase 1	P00558	44854	42000	46%	145
FIBB	fibrinogen beta chain	P02675	56577	55000	63%	300
FIBG	fibrinogen gamma chain	P02679	52106	50000	56%	233
ALBU	serum albumin	P02768	71317	65000	55%	287
CXCL7	platelet basic protein LDGF	P02775	14171	10000	38%	105
ALDOA	fructose-bisphosphate aldolase A	P04075	39720	40000	67%	214
SODM	superoxide dismutase (Mn)	P04179	24878	23000	34%	58
GAPDH	glyceraldehyde-3-phosphate dehydroge-nase	P04406	36070	35000	29%	58
GELS	gelsolin, actin-depoly-merizing factor	P06396	86043	66000	16%	59
ATPB	ATP synthase subunit beta	P06576	56525	53000	60%	225
ENOA	alpha-enolase	P06733	47350	50000	61%	278
TPM3	tropomyosin alpha-3	P06753	32856	33000	30%	75
LDHB	L-lactate dehydro-genase beta chain	P07195	36769	38000	67%	211
PDIA1	protein disulfide-isomerase A1	P07237	57480	55000	38%	172
TUBB	tubulin beta-2 chain	P07437	50095	56000	67%	282
PROF1	profilin-1	P07737	15085	15000	64%	90
HS90A	heat shock protein HSP 90-alpha	P07900	84875	90000	34%	169
ITA2B	integrin alpha IIb	P08514	114460	10500 0	31%	214
ANXA5	annexin V	P08758	35840	27000	30%	62
TPM1	tropomyosin 1 alpha	P09493	32746	38000	25%	78
THIO	thioredoxin	P10599	11884	13000	49%	51
GRP78	78 kDa glucose-regulated protein	P11021	72402	72000	53%	295
HSP7C	Heat shock cognate 71 kDa protein	P11142	71082	68000	58%	264
ACTN1	alpha-actinin-1	P12814	103563	50000	32%	190
PLSL	plastin-2	P13796	70684	64000	48%	281
VINC	vinculin	P18206	124161	66000	34%	288
FLNA	filamin-A	P21333	283192	42000	10%	79
CALR	calreticulin	P27797	48283	40000	23%	70
PSB4	proteasome subunit beta type 4	P28070	29242	28000	28%	54
TKT	transketolase	P29401	68519	67000	24%	75
PDIA3	protein disulfide-	P30101	57146	60000	62%	319

	isomerase A3					
GDIA	Rab-GDI-alpha	P31150	51177	55000	41%	132
MYH9	myosin-9	P35579	227515	60000	10%	71
TALDO	transaldolase	P37837	37688	38000	28%	76
CAZA2	F-actin capping protein alpha-2	P47755	33026	35000	63%	167
LIMS1	LIM and senescent cell antigen-like-contain-ing domain protein 1, protein PINCH	P48059	38977	35000	37%	88
GDIR1	Rho GDP-dissociation inhibitor 1	P52565	23119	28000	47%	103
GDIR2	Rho GDP-dissociation inhibitor 2	P52566	22900	28000	48%	88
ILK	integrin-linked protein kinase 2	P57043	51899	50000	26%	91/90
TPIS	triosephosphate isomerase	P60174	26807	28000	62%	155
ARP3	actin-like protein 3	P61158	47666	50000	64%	275
CALM	calmodulin	P62158	16696	16000	33%	64
GRB2	growth factor receptor-bound protein 2	P62993	25304	28000	29%	77
HBB	hemoglobin subunit beta	P68871	15971	8000	60%	104
STXB2	syntaxin-binding protein 2	Q15833	66853	66000	22%	65
UGPA	UDP-glucose pyro-phosphorylase 2	Q16851	56945	53000	15%	72
SH3L3	SH3 domain-binding protein 1	Q9H299	10488	5000	46%	81
TBA8	tubulin alpha 8	Q9NY65	50746	18000	24%	55
TLN1	talin1	Q9Y490	271766	40000	8%	61
